# The Challenge and Opportunity of Pediatric Antimicrobial Stewardship in Low Resource Settings

**DOI:** 10.1093/tropej/fmz079

**Published:** 2019-12-04

**Authors:** Pui-Ying Iroh Tam

**Affiliations:** Paediatrics and Child Health Research Group, Malawi-Liverpool Wellcome Trust Clinical Research Programme, Blantyre, Malawi & Liverpool School of Tropical Medicine, Liverpool, UK

When penicillin was first discovered by Alexander Fleming in 1928, by the time of its distribution for therapeutic use in the 1940s for treatment of such diseases as syphilis, an almost incurable disease appeared now to be curable. Indeed, for a disease that had for decades been treated with inorganic mercury salts and arsenic compounds, there was optimism that drugs used for so long ‘without any indications of an increased incidence of arsenic-resistant infections, … gives grounds for hoping that the widespread use of penicillin will equally not result in an increasing incidence of infections resistant to penicillin [1]’.

However, while syphilis remains to this day exquisitely sensitive to penicillin that has not been the case for other bacterial pathogens. *Staphylococcus aureus* was controlled by penicillin for only a short period in the 1940s before penicillinases rendered the drug ineffective. As an early alternative to penicillin, erythromycin was introduced for treatment in Boston City Hospital in the early 1950s, but had to be withdrawn within a year, after 70% of all *S. aureus* isolates became resistant. The first designer antiresistance antibiotic, methicillin, was introduced in 1959 as a defense against staphylococcal penicillinases; however, within 3 years methicillin-resistant *S. aureus* (MRSA) isolates appeared [2].

The spread of antimicrobial resistance (AMR) has been particularly marked among Gram-negatives, specifically *Enterobacteriaceae*. Trends in bloodstream infections in a tertiary hospital in Malawi, with quality-assured microbiology laboratory capacity for over 20 years and continuous surveillance throughout that period, documented an increase in MRSA from 7.7% to 18.4% between 1998 and 2016. However, this increase was slight compared to an increase in extended spectrum beta-lactamase resistance from 2003 to 2016 among *Escherichia coli* of 0.7–30.3%, and from 11.8% to 90.5% among *Klebsiella* spp. [3]. When trends in bloodstream infections were evaluated by age groups, the under 5 years had consistently one of the highest incidence for all pathogens amongst all age groups, with the exception of yeast.

Further evaluation of AMR in this age group identified that resistance to empiric first-line antimicrobials was most marked among young infants ≤60 days, with an increase from 7.0% to 67.7% over 20 years, compared to an increase from 3.4% to 30.2% for children ≤5 years [4]. Among children ≤5 years, *Klebsiella* spp. resistance to first-line antimicrobials increased from 5.9% to 93.7% between 1998 and 2017; sample sizes were not large enough to evaluate this for young infants [4].

The recognition of emergence of AMR is not new, particularly as an adverse effect of food animal growth-promoting antibiotics [5]. The Swann report in 1969 recommended the banning of antibiotics for non-therapeutic use in animals and agriculture [6], although to this day this has been impossible to enforce in many countries. In January 1981, the American clinician-scientist Stuart Levy convened a meeting in the Dominican Republic and produced a joint ‘Statement Regarding Worldwide Antibiotic Misuse’ that was signed by 147 scientists from 27 countries (although over half were from the United States). The statement cautioned against: non-prescription antibiotic use, use of agribusiness growth promoters, continued antibiotic usage when not effective or required, overpromotion of antibiotics as wonder drugs and marketing of particular antibiotics differently in different parts of the world [6]. Their subsequent formation of the Alliance for the Prudent Use of Antibiotics aimed to draw the attention of clinicians, pharmaceutical industry and global foundations and public health groups. However, without federal interest or attention paid to this issue [6, 7], gains made were modest.

It was not until the 1990s that international organizations such as the WHO fully engaged with the issue of antibiotic resistance. A series of working groups and meetings were held throughout the decade, culminating in a ‘Global Strategy for Containment of Antimicrobial Resistance’ report that was released in 2001. This report noted the direct health effects of AMR, but also noted the broader economic and national security implications of widespread resistance [8]. However, by 2013, members of the Center for Disease Dynamics, Economics, and Policy, lamented that ‘coordinated action is largely absent, especially at the political level, both nationally and internationally [9]’. The Chief Medical Officer of Great Britain, Dame Sally Davies, issued a report in 2009 titled ‘Infections and the Rise of Antimicrobials’ that described the threat faced by AMR to have ‘the potential to be as important as global warming in terms of its impact on health [10]’. There was also growing recognition that AMR is a problem that interfaces with many sectors of society, from the environment, to animal health, agriculture and aquaculture, to human health; therefore, tackling this problem requires a one health approach.

From the human health perspective, antibiotic usage has been well correlated with resistance. A 7-year survey of the relationship between antibiotic susceptibility and usage in a French hospital documented significant correlations between increase in antibiotic use and decrease in susceptibility [11]. Therefore, antimicrobial stewardship programs (ASPs) have long been accepted as a modality to address AMR and patient care and has been listed as a key component of the WHO Global Action Plan to combat AMR [12]. In high resource settings, ASPs have been associated with reduced costs, decreased hospital length of stay, and a reduction in burden of AMR while maintaining/improving patient outcomes [13].

The challenge of antimicrobial stewardship in low resource settings is obvious, with the scarcity of human and material resources, and limited antibiotics as well as limited access to antibiotics driving inappropriate use, the limited diagnostic capacity, weak data management systems, inexperience with data analysis and lack of trained personnel. In low resource settings, these factors result in three times higher infection density in adult intensive care units (ICUs) and up to nine times higher estimated infection density in neonatal ICUs [14], compared to high resource settings.

However, therein also lies opportunities for pediatric antimicrobial stewardship research. WHO guidelines for Integrated Management of Childhood Illness is based on limited evidence, particularly from low- and middle-income countries (LMICs). However, the AFRINEST group evaluated simplified regimens for treatment of infants with clinical signs of severe infection conducted trials in DR Congo, Nigeria and Kenya, and demonstrated that three simplified regimens, including the use of oral antimicrobials, were as effective as injectable procaine benzylpenicillin-gentamicin for 7 days [15]. Given that outcomes using simplified regimens were as effective, these data pose an opportunity to evaluate in low resource settings whether outpatient use of simplified regimens can reduce unnecessary hospitalizations without compromising patient outcomes, and thereby reduce incidence of hospital-acquired infection and consequent rates of AMR.

Future research in antimicrobial stewardship should be focused on: (i) refining antimicrobial stewardship in the absence of reliable diagnostic and laboratory capacity, including the use of clinical exam and basic laboratory tests to optimize detection and therapy for infection; and evaluating safe de-escalation of therapy in the absence of reliable microbiology and laboratory data; (ii) optimizing diagnostic stewardship, including the assessment of whether use of adjunctive diagnostic tests, such as C-reactive protein and procalcitonin, as demonstrated in high-income countries, leads to more appropriate antimicrobial management; and (iii) Evaluating the effect of simplified antimicrobial regimens on reducing unnecessary hospitalizations and consequent reduction of AMR, while preserving patient health and outcomes. Coordinated engagement from clinicians, public health and policy makers is required if sustainable solutions are sought.

AMR is a global issue, and low resource settings carry a disproportionate burden of AMR, which is significantly associated with mortality [16]. The fact that there has been no major discovery of a new antibiotic in the last 30 years means that all settings must be committed to using their available antimicrobials judiciously. In low resource settings, this provides a unique opportunity to evaluate antimicrobial stewardship management strategies, and to assess the consequent impact of these interventions on patient outcomes and rates of AMR. Children and young infants, among whom rates of infections and associated AMR are highest, would be the rational population to target with such interventions. In this way, low resource settings can set an example for antimicrobial stewardship for the rest of the world.

The world is watching.
